# Activity–Inactivity Patterns, Screen Time, and Physical Activity: The Association with Overweight, Central Obesity and Muscle Strength in Polish Teenagers. Report from the ABC of Healthy Eating Study

**DOI:** 10.3390/ijerph17217842

**Published:** 2020-10-26

**Authors:** Magdalena Górnicka, Jadwiga Hamulka, Lidia Wadolowska, Joanna Kowalkowska, Eliza Kostyra, Marzena Tomaszewska, Jan Czeczelewski, Monika Bronkowska

**Affiliations:** 1 Department of Human Nutrition, Institute of Human Nutrition Sciences, Warsaw University of Life Sciences (SGGW-WULS), Nowoursynowska Str 159C, 02-776 Warsaw, Poland; magdalena_gornicka@sggw.edu.pl; 2 Department of Human Nutrition, Faculty of Food Sciences, University of Warmia and Mazury in Olsztyn, Pl. Cieszynski 1, 10-718 Olsztyn, Poland; lidia.wadolowska@uwm.edu.pl (L.W.); joanna.kowalkowska@uwm.edu.pl (J.K.); 3 Department of Functional and Organic Food, Institute of Human Nutrition Sciences, Warsaw University of Life Sciences (SGGW-WULS), Nowoursynowska Str 159C, 02-776 Warsaw, Poland; eliza_kostyra@sggw.edu.pl; 4 Department of Food Gastronomy and Food Hygiene, Institute of Human Nutrition Sciences, Warsaw University of Life Sciences (SGGW-WULS), Nowoursynowska Str 159C, 02-776 Warsaw, Poland; marzena_tomaszewska@sggw.edu.pl; 5 Faculty of Physical Education and Health in Biała Podlaska, Josef Piłsudski University of Physical Education in Warsaw, Akademicka Str 2., 21-500 Biała Podlaska, Poland; jan.czeczelewski@awf-bp.edu.pl; 6 Department of Human Nutrition, Faculty of Biotechnology and Food Science, Wroclaw University of Environmental and Life Sciences, Chelmońskiego Str 37, 51-630 Wroclaw, Poland; monika.bronkowska@upwr.edu.pl

**Keywords:** screen time, physical activity, activity pattern, inactivity pattern, adiposity, BMI, WHtR, muscle strength, Polish teenagers

## Abstract

Today, the time spent actively is increasingly being replaced by screen-based media, although in some teenagers, a high level of physical activity (PA) and longer time spent in front of a screen (screen time, ST) may coexist as a mixed behavioral pattern. This study aimed to examine the association of the pattern created as activity (low/high ST with high PA) and inactivity patterns (low/high ST with low PA) with overweight, central obesity, and muscle strength in Polish teenagers taking into consideration socioeconomic and demographic factors. Cross-sectional data were collected from elementary school children (*n* = 1567), aged 11–13 years. Height, weight, waist circumference, and handgrip strength were measured. Body mass index (BMI) was calculated as the overweight measure, and the waist-to-height ratio (WHtR) was calculated as the central obesity measure. Data on ST, PA, socioeconomic status, demographics, and nutrition knowledge were collected by a questionnaire. Activity–inactivity patterns were defined by an a priori approach. Multivariate logistic regression modelling was applied. The most active pattern (lowST-highPA) was found in 17% of the total sample. Teenagers with the most inactive pattern (highST-lowPA) had over four times higher chance of general overweight. No association between WHtR ≥0.5 and highST-highPA pattern was found. Higher muscle strength (>1 SD) was associated only with high physical activity. Urban residence or lower socioeconomic status increased adherence to the most inactive pattern. From a public health perspective, implementing interventions promoting active patterns in 11–13-year-old teenagers is important for obesity prevention and enhanced physical fitness, especially in girls, teenagers living in urban areas, and from families with lower socio-economic status.

## 1. Introduction

Currently, obesity, not only in adults but also in children and adolescents, is one of the most serious global problems affecting every country in the world [1]. Nearly one out of six children is overweight or obese in Organisation for Economic Cooperation and Development (OECD) countries (of which Poland has been a member since 1996). In the last four decades, the prevalence rates for obesity have risen ten times and it is assumed that the current trend continues [2]. In Polish children, both general and abdominal obesity have increased significantly over the past 50 years, with a tendency toward central obesity [3].

The increase in obesity occurrence is associated with technological progress and socio-economic development, which has created the so-called “obesogenic environment”. It is well-documented that the increase in obesity occurrence, regardless of the region of the world [4,5,6,7,8], is associated with more sedentary behaviors causing lower energy expenditure and an increase in unhealthy dietary behaviors, such as the consumption of sugar-sweetened beverages and discretionary snacks [9,10]. In recent years, we have seen the increase in access to electronic equipment, including televisions, computers, smartphones, and other mobile devices. This has resulted in a long time spent sitting in front of a screen (screen time, ST), also among children [8]. Furthermore, technological changes in transport (more frequent use of a car, bus, tram, metro/subway/underground), household equipment, and new forms of spending free time with the use of screen devices, has reduced body energy expenditure [11]. Hence, prolonged screen time combined with sitting time may decrease time spent in physical activity (PA), e.g., in the open air or in a sports hall, which could increase the occurrence of obesity and other non-communicable diseases, including diabetes type 2, hypertension, dyslipidaemia or musculoskeletal disorders (especially osteoarthritis), and worsening mental health and social well-being [4,12,13].

In turn, physical activity plays a protective role against excessive body fat mass [14,15] and positively correlates with health indicators such as health status, self-image, quality of life and family and peer relationship [16], as well as healthy child development [17,18]. Moreover, vigorous, systematic PA determines muscle strength [19] and is positively associated with muscular fitness [20] and with muscle mass [21]. Unfortunately, research from recent years suggests that the recommended level of physical activity was not achieved by 80% of children and teenagers in 105 countries worldwide [17].

According to the World Health Organization (WHO) [22], as well as Polish recommendations [23], children and adolescents should be physically active for at least one hour of moderate to vigorous PA per day and vigorous-intensity PA at least three times per week. Also, Polish recommendations for children and adolescents regarding ST, including television watching, using a computer, etc. [23], are consistent with the worldwide applied recommendations of the American Academy of Pediatrics (AAP) [24]. Today, the time spent actively is increasingly being replaced by screen-based media, although in some teenagers, a high level of physical activity (PA) and longer time spent before a screen (screen time, ST) may coexist as a mixed behavioral pattern [25].

Since scientific evidence appears inconclusive and there are no studies in the Polish population of this group of children, the current study aimed to examine the association of the pattern created as activity (low/high ST with high PA) and inactivity patterns (low/high ST with low PA) with overweight, central obesity, and muscle strength in Polish teenagers taking into consideration socioeconomic and demographic factors. It was hypothesized that inactivity patterns can increase the occurrence of obesity and low muscle strength in Polish teenagers, and it can be associated with socio-demographic factors.

## 2. Materials and Methods

### 2.1. Ethical Approval

The study was approved by the Bioethics Committee of the Faculty of Medical Sciences, the University of Warmia and Mazury in Olsztyn, on 17 June 2010 (resolution no. 20/2010) and conducted in accordance with the principles of the Declaration of Helsinki. Informed written consent was obtained from parents or legal guardians of teenagers.

### 2.2. Study Design and Participants

**Figure 1:**
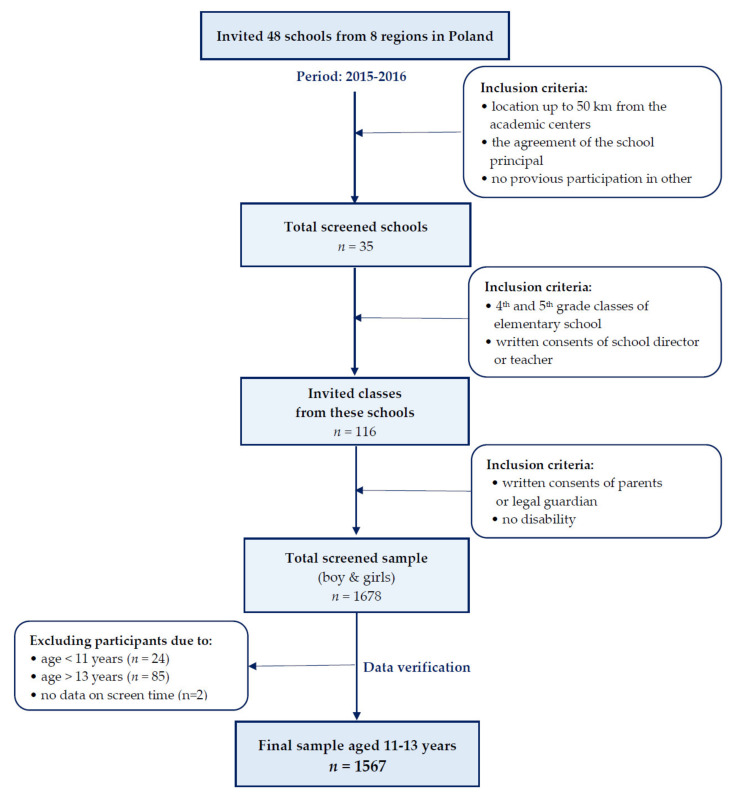
Flowchart of study sample collection.

To achieve the proposed objectives, a cross-sectional study was conducted of the “ABC of Healthy Eating” national multicenter project. Data were collected (in 2015–2016) by researchers from eight academic centers in Poland. Children were recruited among students aged 11–13 years in 35 elementary schools covering the entire territory of Poland. A convenient sample selection was applied. More details on the study design, methods, sample selection (collection) and early findings of the above-mentioned study were described in previous papers [26,27,28] and presented in a flowchart of this study (Figure 1). Finally, the study included 1567 teenagers aged 11–13 years—760 boys (48.5%) and 807 girls (51.5%).

Data related to socioeconomic and demographic factors, nutrition knowledge and lifestyle characteristics, including usual frequency consumption for breakfast, school meal, and nine food items, as well as screen time and total physical activity, were collected with a short-form of a multi-component questionnaire (acronym: SF-FFQ4PolishChildren) developed by Kowalkowska, Wadolowska, and Hamulka for the “ABC of Healthy Eating” project. A reproducibility procedure of the SF-FFQ4PolishChildren (short form of FFQ) and more details on this questionnaire and study were described previously [29].

### 2.3. Measures

In this study, given that ST and PA are interrelated, activity-inactivity patterns were defined by an a priori approach using data on ST and physical PA for integrative classification.

#### 2.3.1. Screen Time (ST)

Screen time was assessed using the question: How much time do you spend watching TV or in front of a computer on an average day of the week? It was explained to participants that all time spent in a sedentary manner should be considered; however, time spent with smartphones (e.g., while walking) should not be considered. The respondents chose one of six categories (hours/day): <2; 2 to <4; 4 to <6; 6 to <8; 8 to <10; ≥10. Response categories of ST were combined into three categories (h/day): <2; 2 to 4; ≥4. Considering the recommended daily maximum of two hours of sedentary screen time for children and teenagers as a reference [23,24,30,31], ST <2 h/day was defined as low, and ≥4 h/day was defined as high.

#### 2.3.2. Physical Activity (PA)

PA was assessed using two questions about the level of PA at school: How would you describe your physical activity in school, and during leisure time, how would you describe your physical activity during your time off (after classes, on weekends)? The respondents chose one of three categories describing their physical activity at school and during leisure time: low, moderate, or vigorous. Examples for each category of PA were provided to choose from. More details on this questionnaire part have been provided in previous papers [26,29]. Finally, after combining categories of responses, three total PA levels—low PA, moderate PA, and high PA—were created [26].

#### 2.3.3. Activity–Inactivity Patterns

Patterns were defined by an a priori approach using previously developed categories for ST and PA (Table 1), and based on a definition of inactivity as an insufficient level of activity [32]. For further analysis, four main patterns were selected: highST-lowPA (the most inactive pattern), lowST-lowPA, highST-highPA, and lowST-highPA (the most active pattern). Each participant was allocated to one of four patterns. All categories with moderate ST and/or moderate PA were combined as “other” but were not included in further analysis. Based on recommendations regarding PA and ST, the lowST-highPA pattern was assumed as a reference [33].

#### 2.3.4. Anthropometric Data

Height, weight, waist circumference, and handgrip strength were directly measured by qualified researchers during an in-school visit according to International Standards for Anthropometric Assessment [34]. Height (to the nearest 0.1 cm) was measured using a SECA 220 portable telescopic measuring rod (Hamburg, Germany). Weight (to the nearest 0.1 kg) was measured using an electronic digital scale (SECA 799, Hamburg, Germany) and waist circumference by a stretch-resistant tape that provides a constant 100 g tension (SECA 201, Hamburg, Germany). All measurements were performed under strictly standardized conditions (between 08:00 and 12:00) using the same device to avoid inter-observer and inter-device variability and after explaining the procedure to each child. Measurements were taken twice in light clothing and without shoes, and the averages were calculated.

To identify general overweight, gender-and age-related body mass index (BMI) was applied with cut-offs as BMI ≥ 25 kg/m^2^, according to the International Obesity Task Force (IOTF) [35]. To identify central obesity, measurement of the waist-to-height ratio (WHtR) with a cut-off point of 0.5 was used [36].

Handgrip strength (kg) was measured twice for the dominant hand, according to the respondent’s declaration (right or left), using a hydraulic hand dynamometer (manufacturer; SAEHAN Corporation, Masan, Korea). For further analyses, the average value of two measurements was taken. The z-score of handgrip strength (z-HGS) was calculated based on the authors’ own database to achieve a mean equal to 0 and standard deviation (SD) equal to 1. Based on variable distribution, the z-score was categorized as follows: <−1 SD as lower muscle strength; −1 to 1 SD as normal; and >1 SD as higher muscle strength [26].

#### 2.3.5. Socioeconomic and Demographic Data

The demographic predictors of activity-inactivity patterns included gender, age, and residence (rural or urban). The socioeconomic status was determined using the Family Affluence Scale (FAS) and included four questions (about the family car, one’s own bedroom, family holidays, and the number of computers/laptops/tablets) prepared by the Polish team in the international HBSC study [37]. Points were assigned to each answer and summed up for each respondent (range 0–7). Based on quartile distribution, the respondents were divided into three FAS categories labelled as low (0–4 points; <25th quartiles), moderate (5–6 points), and high (7 points; ≥75th quartiles).

#### 2.3.6. Nutrition Knowledge Score

This score was determined based on eighteen questions, developed by Whati et al. [38] and adapted to Polish conditions and education [26]. The correct answer scored 1 point, and wrong or 

“I don’t know” answers or missing answers scored 0 points. After summing up (0–18 points) nutrition knowledge score was calculated for each participant.

### 2.4. Statistical Analysis

Variables were presented as a sample percentage (%) or mean and standard deviation. The differences between groups were verified by Chi^2^ Pearson test for categorical variables or Kruskal–Wallis test for continuous variables.

A multivariate logistic regression modelling was applied to assess:

1. The chance to fall in the category of central obesity or overweight or higher muscle strength. Activity (highST-highPA) and inactivity patterns (lowST-lowPA, highST-lowPA) were used as predictors, while the lowST-highPA pattern was used as a reference.
2. The adherence to activity–inactivity patterns by socioeconomic and demographic factors, in respect to a referent lowST-highPA pattern, the following categorical variables were used as predictors (independent variables): gender (girls, reference: boys), age (12 or 13 years, reference: 11 years), residence (urban, reference: rural), and Family Affluence Scale (moderate or high, reference: low).

The odds ratios (ORs) and 95% confidence interval (95% CI) were calculated. The ORs were adjusted (OR_A_) for gender, age (years), residence (two categories), FAS (points), and nutrition knowledge score (in points, excluding the modelled variable from the confounders set, respectively). The significance of ORs was assessed by Wald’s statistics. For all tests, *p* < 0.05 was considered significant. All statistical analyses were performed using STATISTICA software (version 12.0 PL; StatSoft Inc., Tulsa, OK, USA; StatSoft, Krakow, Poland).

## 3. Results

The demographic and socioeconomic characteristics of the study population are presented in Table 2. The sample consisted of 48% boys and 52% girls. Most of the teenagers were aged 12 years (74%), had urban residence (60%) and about half came from families with moderate FAS. Overall, 46% of teenagers declared ST < 2 h/day and 31% indicated high PA (Table 2). Over 42% of boys and 50% of girls reported ST < 2 h/day, while 23% and 16% of boys and girls, respectively, reported high ST (≥4 h/day). High PA was found in 37% of boys and 26% of girls (Table 2). About 46% of teenagers reported vigorous PA at school and 50% declared vigorous PA at leisure time (Table S1). More boys than girls had ST ≥ 4 h/day (Table 2) and high PA, vigorous PA at school and at leisure time (Table S2). Over 21% of teenagers with urban residence and 16% of with rural reported ST ≥ 4 h/day. A lower percentage of teenagers with high FAS compared with low FAS declared ST ≥ 4 h/day, (17% vs. 23%), low PA in total (5% vs. 15%) (Table 2), low PA at school (4% vs. 8%) or at leisure time (6% vs. 15%) (Table S1). Only about 17% of teenagers represented the most active pattern (lowST-highPA), which included more boys (19% vs. 15% of girls), teenagers with high FAS (20% vs. 12% of with low FAS), and with a higher value of Nutrition Knowledge Score (Table 3).

Overweight was found in 25% of teenagers, while central obesity in 12% (Table 4). Nearly 40% of teenagers with highST-lowPA pattern and 34% with a lowST-lowPA pattern were overweight. The percentage of teenagers with central obesity (23.4%) was the highest in teenagers with a lowST-lowPA pattern, while the lowest (4%) was found in teenagers with the most active pattern (lowST-hihg PA). Over 32% of teenagers with ST ≥ 4 h/day and 42% with low PA were overweight, while 18% and 22%, respectively, had central obesity (Table 4). Only about 8% of teenagers with low PA had higher muscle strength, compared with 17% with high PA (Table 4).

**Figure 2:**
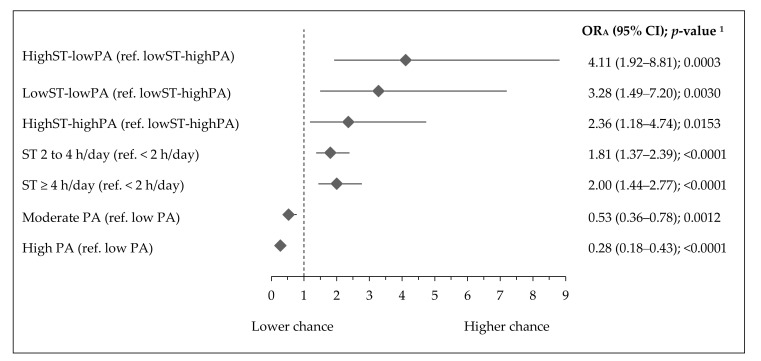
Association of activity–inactivity patterns, ST, and PA with overweight in Polish teenagers (ref. normal weight). Notes: ST—screen time; PA—physical activity; overweight and normal weight identified as gender-age-related-BMI according to international standards [35] as follows: overweight BMI ≥ 25 kg/m^2^, normal weight BMI = 18.5 to 24.9 kg/m^2^; OR_A_—odds ratio adjusted for gender, age (years), residence (categorical variable), Family Affluence Scale (points), and Nutrition Knowledge Score (points); 95% CI—confidence interval; ^1^ Wald test significance level.

**Figure 3:**
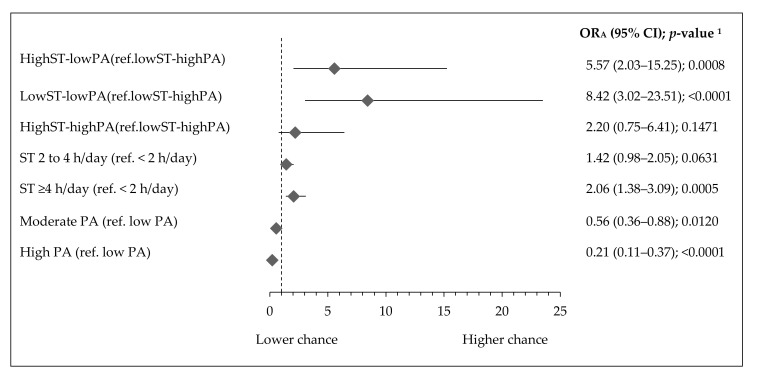
Association of activity–inactivity patterns, ST, and PA with central obesity in Polish teenagers (ref. lack of central obesity). Notes: ST—screen time; PA—physical activity; central obesity identified as waist-to-height ratio ≥0.5; lack of central obesity identified as waist-to-height ratio <0.5 [36]; OR_A_—odds ratio adjusted for gender, age (years), residence (categorical variable), Family Affluence Scale (points) and Nutrition Knowledge Score (points); 95% CI—confidence interval; ^1^ Wald test significance level.

**Figure 4:**
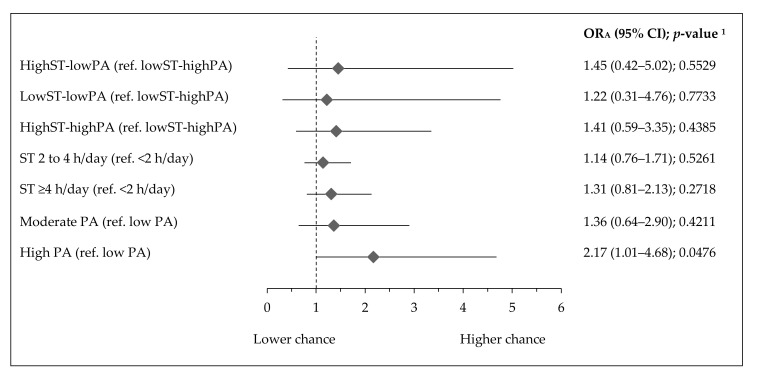
Association of activity–inactivity patterns, ST, and PA with higher muscle strength in Polish teenagers (ref. normal muscle strength). Notes: ST—screen time; PA—physical activity; higher and normal muscle strength identified as z-score handgrip strength as follows: higher >1 SD, normal—1 to 1 SD; OR_A_—odds ratio adjusted for gender, age (years), residence (categorical variable), Family Affluence Scale (points) and Nutrition Knowledge Score (points); 95% CI—confidence interval; ^1^ Wald test significance level.

The associations of activity–inactivity patterns with overweight, central obesity, and muscle strength are shown in Figure 2, Figure 3 and Figure 4. Teenagers with the most inactive pattern (highST-lowPA) had over four times higher chance of overweight (OR_A_: 4.11, 95% CI 1.92–8.81; *p* = 0.0003), while those with the lowST-lowPA pattern had over three times higher chance of overweight (OR_A_: 3.28, 95% CI 1.49–7.20; *p* = 0.003). Teenagers with the highST-highPA pattern had over two times higher chance of overweight (OR_A_: 2.36, 95% CI 1.18–4.74; *p* = 0.0153) in comparison with the reference active pattern (lowST-highPA) (Figure 2). Teenagers with ST >2 h/d had 1.8–2 times higher chance of being overweight (OR_A_: 1.81, 95% CI 1.37–4.74; *p* = 0.012 and OR_A_: 2.00, 95% CI 1.44–2.77; *p* < 0.0001), while teenagers with high PA (OR_A_: 0.28, 95% CI 0.18–0.43; *p <* 0.00001) (Figure 2), with vigorous PA at school (OR_A_: 0.45, 95% CI 0.27–0.75; *p* = 0.002), or with vigorous PA at leisure time (OR_A_: 0.48, 95% CI 0.30–0.78; *p* = 0.0026), (Table S4) had approximately 52–72% lower chance of being overweight.

Teenagers with the lowST-lowPA pattern had over eight times higher chance of developing central obesity (OR_A_: 8.42, 95% CI 3.02–23.51; *p* < 0.0001), while teenagers with a highST-lowPA pattern had over five times higher chance of central obesity (OR_A_: 5.57, 95% CI 2.03–15.25; *p* = 0.0008) (Figure 3). No association between central obesity and the highST-highPA pattern was found. Teenagers with ST ≥ 4 h/day had two times higher chance of central obesity (OR_A_: 2.06, 95% CI 1.38–3.09; *p* = 0.0005), while teenagers with high PA (OR_A_: 0.21, 95% CI 0.11–0.37; *p* < 0.0001) (Figure 3), with vigorous PA at school (OR_A_: 0.38, 95% CI 0.20–0.71; *p* = 0.0023) or with vigorous PA at leisure time (OR_A_: 0.31, 95% CI 0.18–0.51; *p* < 0.00001) (Table S4) had approximately 62–79% lower risk of central obesity.

No association between activity-inactivity patterns with muscle strength was found (Figure 4). Teenagers with high PA had 2.2 times higher chance of having higher muscle strength (OR_A_: 2.17, 95% CI 1.01–4.68; *p* = 0.0476).

**Figure 5:**
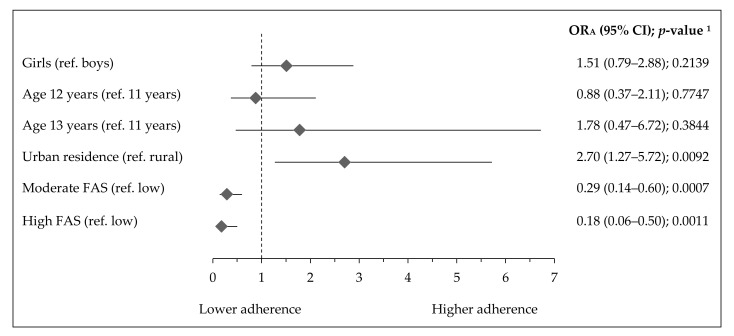
Factors associated with highST-lowPA pattern in Polish teenagers (ref. lowST-highPA pattern). Notes: ST—screen time; PA—physical activity; FAS—Family Affluence Scale; OR_A_—odds ratio adjusted for gender, age (years), residence (categorical variable), FAS (points) and Nutrition Knowledge Score (points) excluding the modelled variable from confounders set, respectively; 95% CI—confidence interval; ^1^ Wald test significance level.

**Figure 6:**
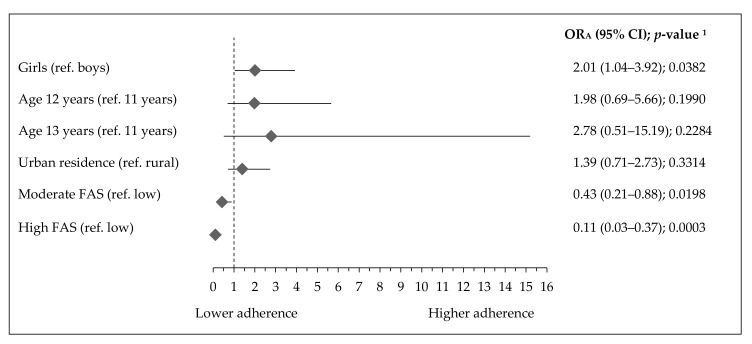
Factors associated with lowST-lowPA pattern in Polish teenagers (ref. lowST-highPA pattern). Notes: ST—screen time; PA—physical activity; FAS—Family Affluence Scale; OR_A_—odds ratio adjusted for gender, age (years), residence (categorical variable), FAS (points) and Nutrition Knowledge Score (points) excluding the modelled variable from confounders set, respectively; 95% CI—confidence interval; ^1^ Wald test significance level.

**Figure 7:**
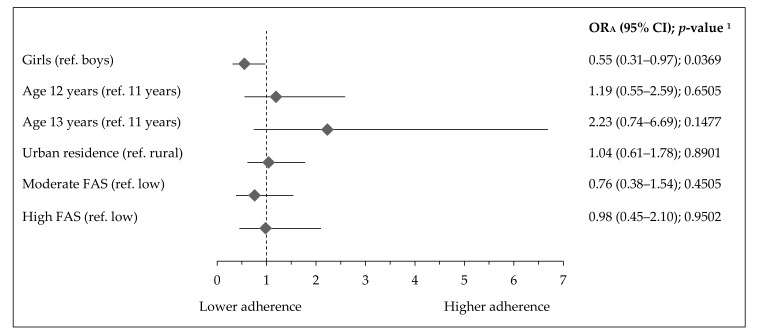
Factors associated with highST-highPA pattern in Polish teenagers (ref. lowST-highPA pattern). Notes: ST—screen time; PA—physical activity; FAS—Family Affluence Scale; OR_A_—odds ratio adjusted for gender, age (years), residence (categorical variable), FAS (points) and Nutrition Knowledge Score (points) excluding the modelled variable from confounders set, respectively; 95% CI—confidence interval; ^1^ Wald test significance level.

Socioeconomic and demographic factors associated with activity-inactivity patterns are presented in Figure 5, Figure 6 and Figure 7. The adherence to the most inactive pattern (highST-lowPA) was positively associated with urban residence (OR_A_: 2.70, 95% CI 1.27–5.72; *p*= 0.0092), while negatively with moderate FAS (OR_A_: 0.29, 95% CI 0.14–0.60; *p* = 0.0007) or high FAS (OR_A_: 0.18, 95% CI 0.06–0.50; *p* = 0.0011) (Figure 5). Girls had a higher adherence to the lowST-lowPA pattern (OR_A_: 2.01, 95% CI 1.04–3.92; *p* = 0.0382), while teenagers with moderate FAS (OR_A_: 0.43, 95% CI 0.21–0.88; *p* = 0.0198) or high FAS (OR_A_: 0.11, 95% CI 0.03–0.37; *p* = 0.0003) had lower adherence (Figure 6). The adherence to the highST-highPA pattern was lower by 45% in girls (OR_A_: 0.55, 95% CI 0.31–0.97; *p* = 0.0369) (Figure 7).

The lower chance of high ST (≥4 h/day) belonged to girls (OR_A_: 0.62, 95% CI 0.47–0.82; *p* = 0.0008) and teenagers with moderate FAS (OR_A_: 0.70, 95% CI 0.50–0.98; *p* = 0.0351), while higher chance with urban residence (OR_A_: 1.45, 95% CI 1.08–1.93; *p* = 0.0122) (Table S5). Teenagers with high FAS had a higher chance of high PA in total, vigorous PA at school or PA at leisure time (OR_A_: 3.53, 95% CI 1.97–6.31; *p* < 0.0001, and OR_A_: 2.70, 95% CI 1.38–5.31; *p* = 0.0038, and OR_A_: 3.06, 95% CI 1.79–5.24; *p* < 0.0001, respectively). In turn, female gender or urban residence lowered the chance of high total PA (OR_A_: 0.67, 95% CI 0.46–0.99; *p* = 0.043, and OR_A_: 0.62, 95% CI 0.41–0.92; *p* = 0.0171). Moreover, urban residence lowered the chance of vigorous PA at leisure time (OR_A_: 0.63, 95% CI 0.43–0.92; *p* = 0.0174) (Table S5).

A similar association between activity–inactivity patterns, ST, PA, and overweight, central obesity, and muscle strength (Table S6) and socioeconomic and demographic factors were found in crude models (Table S7). Those results are presented in Supplementary Materials.

## 4. Discussion

According to our knowledge, this study is the first study investigating the association of activity–inactivity patterns with obesity-related measures BMI and WHtR as well as with muscle strength in young adolescents living in Poland. The study revealed that the most active pattern, that combined the low screen time with high physical activity, was identified in almost 17% of teenagers. However, considering screen time or physical activity alone, over 40% of teenagers reported low screen time and over 30% reported high physical activity. This clearly shows that separate studies on screen time or physical activity level could also lead to overestimation. The current results confirmed the hypothesis that lifestyle behaviors of the majority of Polish teenagers can increase their obesity risk. It was found that, regardless of the time spent looking at a screen, low levels of physical activity were associated with higher chances of being overweight or having central obesity. Moreover, central obesity (assessed by WHtR) was more strongly associated with inactivity patterns (low physical activity, regardless of screen time), and no association was found with high screen time-high physical activity patterns. Higher muscle strength was associated only with high physical activity. Urban residence or lower family affluence increased adherence to the most inactive pattern. Girls had higher adherence to a pattern combining low physical activity with low time spent before a screen.

These findings indicate the need to change lifestyle behaviors in adolescents to reduce the prevalence of metabolic diseases in adulthood, often associated with excessive body weight. In previous studies integrative classification of high screen time and low physical activity [39] or moderate-to-vigorous physical activity and sedentary behavior [40] was used to examine their associations with health indicators. Results showed that adolescents with sedentary behaviors had higher odds of overweight and abdominal obesity [39] or higher cardiometabolic risk [40]. Cristi-Montero et al. [40] concluded that meeting the current physical activity recommendations and reducing time spent sedentary in European adolescents was beneficial for cardiometabolic health, which also depends on obesity. Sedentary behaviors are associated with serious physical health problems, primarily in the context of obesity [41]. Currently, new technologies favor a sedentary lifestyle and thus increase the obesity risk of adolescents. Despite knowledge of the increasing prevalence of obesity and the role of screen-based activities as a major contributor to the obesity epidemic, there is a lack of practical guidelines for parents, teachers, and practitioners to limit digital technologies and online activities in everyday life [41].

Our findings on strong associations between central obesity and inactivity patterns, and not with the high screen time–high physical activity pattern, extend knowledge about obesity risk factors in the developing population. The association between physical activity or screen time and obesity are more often investigated using body mass index (BMI) [42,43,44], although waist-to-height ratio (WHtR) may be a better marker of adiposity in children [45,46]. Central obesity more strongly correlates with metabolic risk factors and is a better predictor of cardiometabolic risk in children [47]. Comparing those results with general overweight assessed by BMI, central obesity was more strongly associated with inactivity patterns. This finding is not entirely consistent with the results of Engberg et al. who studied Finnish children aged 11 years. They analyzed the relationship between screen time and BMI and WHtR and reported similar associations of TV viewing and computer use with BMI and WHtR [48]. However, it is important to note that the current study concerned patterns composed of physical activity and time spent before a screen.

There was no association between muscle strength and activity-inactivity patterns. Higher muscle strength was only associated with high total physical activity or vigorous physical activity at school. In the current study, we used handgrip strength as an indirect measure of muscle strength. It should be noted that although handgrip is a measure of upper limb strength, it was previously found that it correlates well with other measures of upper and lower body strength [49], muscle mass, and bone mineral density in children and adolescents [49,50]. Muscle strength in teenagers is more often investigated concerning physical activity and a positive relationship between them has been reported [19]. Results of studies examining muscle strength and its association with screen time are limited and inconsistent, although inverse associations between viewing television and sedentary behavior and physical fitness have generally been reported [51].

The current findings highlight the crucial role of activity pattern promotion, including an increase in physical activity level in obesity prevention and higher muscle strength. Teenagers with high physical activity at school and at leisure time had a lower chance of being overweight and central obesity. Thus, according to the findings of the current study, meeting physical activity recommendations should be the primary public health strategy more than reducing screen time. Reducing only screen time may be insufficient to prevent adiposity because it may not increase the time spent on physical activity and, in consequence, the risk of overweight may increase [40,52]. To improve the current situation, it seems that the best place to implement programs related to nutrition and lifestyle could be in schools. School-based interventions, according to research, have the potential and effectiveness of programs by targeting a variety of health behaviors [53,54]. As indicated by Throuvala et al. [41] there is an urgent need for more integrated, health-promoting prevention programs in the school environment, targeting a change from sedentary to an active lifestyle.

In this study, it was hypothesized that inactivity patterns, would be associated with socio-demographics. The results indicated that girls had higher adherence than boys to the low screen time-low physical activity pattern but had lower adherence to the high screen time–high physical activity pattern. These results concerning lifestyle components and gender are in line with previously reported results that screen time and physical activity are gender-dependent. Although boys are more engaged in higher levels of screen time [55,56,57,58,59], they are also more positive about physical activity than girls [60]. Boys have more opportunities to be active in school, out of school and outdoors, and they are more encouraged to practice sports [61,62] through a wider offer of sports activities. Furthermore, the gender differences are explained sociologically and are associated with the changing but still existent division of social roles in adult life [61]. Girls spend less time using screen devices, but are also less physically active than boys and are at higher risk of physical inactivity [56,58,63,64,65,66]. These findings confirm that girls should be specifically targeted for physical activity campaigns, both at school and in their leisure time.

Furthermore, the study found that teenagers with lower socioeconomic status had higher adherence to inactivity patterns (low screen time–low physical activity or high screen time–low physical activity). As other authors [67,68,69] have indicated, children from families with low socioeconomic status have a higher risk of unhealthier lifestyle, including more time spent watching TV. Family socioeconomic status plays a key role in teenagers’ physical activity [70]. It has also been shown that teenagers from high-status families had better access to sports facilities, exercised more [69] and had better physical fitness [71]. These findings confirm that using public health policy to increase physical activity should also take into account socioeconomic determinants.

The current study also revealed that urban residence was strongly associated with the most inactive pattern in Polish teenagers. Findings by other researchers have been mixed. Some of them show [72,73] that teenagers from urban areas had lower levels of physical activity, while others reported that rural adolescents are more exposed to sedentary behavior [74]. The differences may be due to national environmental factors, e.g., urbanization or annual average national temperature [75] or it may result from social awareness. In addition, the availability of public leisure spaces, such as squares, courts, skate parks, swimming pools and bike paths are important for increasing physical activity. In Poland, challenges include a lack of attractive sports activities in schools and school sports infrastructure (and access to it), as well as the low physical activity of children associated with the route to school [76]. Due to the growing percentage of obese teenagers, it seems important to create favorable conditions to increase physical activity for all children regardless of the place of residence or socioeconomic status and to increase accessibility to free public leisure spaces, especially outdoors.

### Strengths and Limitations

The strengths of the present study include using activity-inactivity patterns concerning general overweight (BMI), central obesity (WHtR), and muscle strength in Polish teenagers. The current study measured the weight, height, waist circumference and handgrip strength and used two obesity-related outcomes to give a broader picture on examining associations. An important strength is the relatively large sample of teenagers from different regions of Poland. The sample size was large enough (above 1500) to determine statistically significant associations that are also meaningful in practice.

Although the sample was not randomly selected, it does widely reflect the sociodemographic status of Polish society, which the authors believe provides a good basis for the creation of such generalizations.

The limitations of the current study also need to be mentioned. First, the main limitation was using self-reported data on screen time and physical activity, although in other studies self-reporting has been found to offer satisfactory reliability in terms of health-related behaviors [77]. It was decided to use this form due to the larger sample size and the children’s age. Moreover, the presence of qualified researchers while filling in the questionnaire allowed the teenagers to clarify any uncertainties. However, incorrect indication of individual categories may result in a non-differentiation error. Second, only television and computer time were considered screen time. Although television is still the dominant media for family time, there is increased exposure and accessibility to mobile screen media devices and active gaming among adolescents. This may also have the potential to increase moderate physical activity [57]. Future research should include evolving forms of non-sedentary games with motion controllers, which may also require body movements and affect the time spent actively. In this study, dietary patterns and food consumption (which also affect body weight) were not taken into account, which should also be considered a limitation. Finally, the cross-sectional design of the study precludes the investigation of casual relationships.

## 5. Conclusions

Inactivity patterns composed of low physical activity (regardless of time spent before a screen) were associated with a higher chance of overweight and central obesity, while no association with muscle strength was found. However, high physical activity, considered alone, in comparison with low physical activity, was negatively associated with general overweight and central obesity and was positively associated with muscle strength. From a public health perspective, implementing interventions promoting active patterns in 11–13-year-old teenagers is important for obesity prevention and enhancing physical fitness. These findings can be used by policymakers to develop guidelines and develop strategies to reduce inactivity for teenagers. Girls, particularly teenagers with urban residence or those with lower socioeconomic status, due to a combination of prolonged screen time with lower levels of physical activity, should be the population targeted by public health efforts to prevent obesity and improve youth well-being. For future research, these findings shed light on the importance of assessing the chance of obesity through an integrative classification of physical activity and time spent before a screen.

## Figures and Tables

**Table 1:** Activity–inactivity patterns categorized as screen time (ST) or physical activity (PA).

Physical Activity	Screen Time			Patterns *
	Low	Moderate	High	
low	**lowST-lowPA**	other	**highST-lowPA** THE MOST INACTIVE PATTERN	**inactive**
moderate	other	other	other	---
high	**lowST-highPA** THE MOST ACTIVE PATTERN	other	**highST-highPA**	**active**

* the main patterns taken into account in further analysis are marked with the bold fonts.

**Table 2:** Characteristics of participants (% N or mean ± SD).

Variables	Total Sample		Screen Time (h/day)			*p*-Value ^2^	Physical Activity			*p*-Value
	N	%	<2	2 to <4	≥4		Low	Moderate	High	
Sample size ^1^	1567		726	539	302		154	923	490	
Sample percentage		100.0	46.3	34.4	19.3		9.8	58.9	31.3	
Gender										
boys	758	48.4	42.1	35.2	22.7	0.0006	10.0	53.3	36.7	<0.0001
girls	809	51.6	50.3	33.6	16.1		9.5	64.2	26.3	
Age (years)										
11	260	16.6	47.3	37.3	15.4	0.4753	8.4	58.2	33.3	0.3168
12	1153	73.6	46.3	33.8	19.9		10.0	58.2	31.8	
13	154	9.8	44.8	33.8	21.4		10.5	65.4	24.2	
Residence										
rural	631	40.3	46.8	37.4	15.8	0.0099	7.9	58.2	33.9	0.0507
urban	936	59.7	46.0	32.4	21.6		11.0	59.4	29.6	
Family Affluence Scale (categories)										
low	381	24.3	40.9	36.5	22.6	0.0293	15.0	58.8	26.2	<0.0001
moderate	781	49.9	49.7	31.6	18.7		9.5	59.2	31.3	
high	403	25.8	45.2	37.5	17.4		5.4	58.2	36.4	
Family Affluence Scale (0–7 points)										
Mean ± SD	1565	99.9	5.4 ± 1.5	5.4 ± 1.5	5.2 ± 1.6	0.1208	4.7 ± 1.8	5.4 ± 1.5	5.5 ± 1.4	<0.0001
Nutrition Knowledge Score (0–18 points)										
Mean ± SD	1566	99.9	6.2 ± 2.8	6.2 ± 2.8	5.5 ± 2.8	0.0003	5.8 ± 2.9	6.0 ± 2.8	6.3 ± 2.9	0.1619

^1^ Sample size may vary in variables due to missing data; ^2^ significance level of Chi^2^ Pearson test for categorical variables or Kruskal–Wallis test for continuous variables.

**Table 3:** Activity–inactivity patterns in participants by variables (% N or mean ± SD).

Variables	*n*	Activity-Inactivity Patterns (N = 1567)				*p*-Value ^1^
		LowST-HighPA	HighST-HighPA	LowST-LowPA	HighST-LowPA	
N	438	261	76	50	51	
% N	28.0	16.7	4.8	3.2	3.3	
Gender						
boys	241	18.6	7.1	2.8	3.3	0.0002
girls	197	14.9	2.7	3.6	3.2	
Age (years)						
11	68	16.9	3.8	1.9	3.5	0.6596
12	332	17.2	4.9	3.6	3.1	
13	38	12.4	6.5	2.6	3.9	
Residence						
rural	171	17.3	5.1	3.0	1.7	0.0914
urban	267	16.3	4.7	3.3	4.3	
Family Affluence Scale (categories)					
low	104	11.8	4.5	5.0	6.3	<0.0001
moderate	218	17.3	4.4	3.5	2.7	
high	116	20.1	6.2	1.0	1.5	
Family Affluence Scale (0–7 points)					
Mean ± SD	438	5.6 ± 1.4	5.5 ± 1.5	4.4 ± 1.9	4.5 ± 1.7	<0.0001
Nutrition Knowledge Score (0–18 points)						
Mean ± SD	438	6.4 ± 2.8	5.4 ± 2.6	6.2 ± 3.0	5.3 ± 2.8	0.0016

N—the sample size; *n*—the subsample number; ^1^ significance level of Chi^2^ Pearson test for categorical variables or Kruskal–Wallis test for continuous variables; ST—screen time, PA—physical activity.

**Table 4:** Distributions (%) of body mass index (BMI) category, central obesity and muscle strength by activity–inactivity patterns, screen time (ST), and physical activity (PA).

Variables	Gender-Age-Related-BMI ^2^			*p*-Value ^5^	Central Obesity ^3^	*p*-Value	Muscle Strength ^4^			*p*-Value
	Underweight	Normal Weight	Overweight				Lower	Normal	Higher	
Sample size ^1^	145	980	368		182		147	892	148	
Sample percentage	9.7	65.6	24.6		12.2		12.4	75.1	12.5	
Activity-inactivity patterns										
lowST-highPA	11.2	77.2	11.6	<0.0001	4.4	<0.0001	9.8	77.8	12.4	0.3367
highST-highPA	8.2	68.5	23.3		8.2		5.3	77.2	17.5	
lowST-lowPA	6.4	59.6	34.0		23.4		22.2	69.4	8.3	
highST-lowPA	6.3	54.2	39.6		20.8		16.7	72.2	11.1	
Screen time (hours/day)										
<2	11.1	70.6	18.3	<0.0001	9.2	0.0004	11.9	76.9	11.2	0.6425
2 to <4	9.2	61.7	29.1		12.9		13.2	74.2	12.7	
≥4	7.3	60.5	32.2		18.2		12.2	73.0	14.9	
Physical activity										
low	5.6	52.1	42.4	<0.0001	22.2	<0.0001	16.5	75.7	7.8	0.0072
moderate	10.2	63.6	26.1		13.8		13.3	75.8	10.9	
high	9.8	73.6	16.6		6.2		9.3	73.9	16.8	

^1^ Sample size may vary in variables due to missing data; ^2^ gender-age-related-BMI categorized according to international standards [35] as follows: underweight BMI < 18.5 kg/m^2^; normal weight BMI = 18.5 to 24.9 kg/m^2^; overweight BMI ≥ 25 kg/m^2^; ^3^ central obesity identified as waist-to-height ratio (WHtR) ≥ 0.5 [36]; ^4^ muscle strength identified as z-HGS (z-score handgrip strength) as follows: lower <−1 SD, normal −1 to 1 SD, higher >1 SD; ^5^ significance level of Chi^2^ Pearson test.

## Supplementary Materials

The following are available online at https://www.mdpi.com/1660-4601/17/21/7842/s1, Table S1. Distributions (%) of variables by physical activity at school or at leisure time (N = 1567); Table S2. Distributions (% of the sample) of physical activity by screen time; Table S3. Distributions (%) of BMI category, central obesity and muscle strength by physical activity at school or at leisure time; Table S4. Association between physical activity at school, physical activity at leisure time and overweight, central obesity or muscle strength (OR_A_); Table S5. Association between physical activity at school, physical activity at leisure time and socioeconomics or demographics (OR_A_); Table S6. Association between activity-inactivity patterns, ST, PA, and overweight, central obesity or muscle strength (crude OR); Table S7. Association between activity-inactivity patterns, ST, PA, and socioeconomics or demographics (crude OR).

## Abbreviations

95% CI	Confidence Intervals
BMI	Body Mass Index
FAS	Family Affluence Scale
HGS	Hand Grip Strength
ORs	Odds Ratios
PA	Physical Activity
SD	Standard Deviation
ST	Screen Time
WC	Waist Circumference
WHtR	Waist-to-Height Ratio
