# Supplementary material for: Activity–Inactivity Patterns, Screen Time, and Physical Activity: The Association with Overweight, Central Obesity and Muscle Strength in Polish Teenagers. Report from the ABC of Healthy Eating Study
Source: Int J Environ Res Public Health. 2020 Oct 26;17(21):7842. doi: 10.3390/ijerph17217842 (PMC7662883; doi:10.3390/ijerph17217842)
Supplement: Supplementary file 1 [file ijerph-17-07842-s001.pdf]

International Journal of Environmental Research and Public Health

MDPI

Supplementary Materials: Activity–Inactivity Patterns, Screen Time, and Physical Activity: The Association with Overweight, Central Obesity and Muscle Strength in Polish Teenagers. Report from the ABC of Healthy Eating Study

Table S1. Distributions (%) of variables by physical activity at school or at leisure time (N = 1567).

Variables	Physical activity at school			p-value[2]	Physical activity at leisure time			p-value
	low	moderate	vigorous		low	moderate	vigorous	
**Sample size[1]**	86	766	715		149	631	787	
**Sample percentage**	5.5	48.9	45.6		9.5	40.3	50.2	
**Gender**								
boys	6.1	43.6	50.4	0.0002	10.0	37.6	52.4	0.1046
girls	5.1	53.9	41.0		9.0	42.9	48.1	
**Age (years)**								
11	5.0	46.7	48.3		8.0	38.3	53.6	
12	5.8	48.0	46.2	0.0862	9.7	40.1	50.2	0.4373
13	4.6	59.5	35.9		10.4	45.5	44.2	
**Residence**								
rural	5.1	49.2	45.7	0.7847	7.4	40.3	52.2	0.0602
urban	5.9	48.7	45.5		10.9	40.3	48.8	
**Family Affluence Scale (categories)**								
low	7.9	53.0	39.1		15.2	41.5	43.3	
moderate	5.4	48.8	45.8	0.0045	8.6	41.2	50.3	<0.0001
high	3.7	45.0	51.2		5.7	37.6	56.7	
**Family Affluence Scale (0-7 points)**								
Mean ± SD	4.9 ± 1.7	5.2 ± 1.6	5.5 ± 1.4	0.0001	4.7 ± 1.8	5.3 ± 1.5	5.5 ± 1.4	<0.0001
**Nutrition Knowledge Score (0-18 points)**								
Mean ± SD	6.2 ± 3.2	6.0 ± 2.7	6.1 ± 2.9	0.6647	5.4 ± 2.8	6.0 ± 2.7	6.3 ± 2.8	0.0014

[1]Sample size may vary in variables due to missing data; [2]significance level of Chi^2 Pearson test for categorical variables or Kruskal-Wallis test for continuous variables.

International Journal of *Environmental Research and Public Health*

MDPI

Table S2. Distributions (% of the sample) of physical activity by screen time.

Variables	Total sample					Boys					Girls				
	N or %	Screen time (h/day) < 2	Screen time (h/day) 2 to <4	Screen time (h/day) ≥4	p-value[2]	N or %	Screen time (h/day) < 2	Screen time (h/day) 2 to <4	Screen time (h/day) ≥4	p-value	N or %	Screen time (h/day) < 2	Screen time (h/day) 2 to <4	Screen time (h/day) ≥4	p-value
**Sample size[1]**	**1567**	726	539	302		758	319	267	172		809	407	272	130	
**Sample percentage**	**100.0**	46.3	34.4	19.3		48.4	20.4	17.0	11.0		51.6	26.0	17.4	8.3	
**Physical activity**															
low	153	3.2	3.3	3.3		76	2.8	4.0	3.3		77	3.6	2.7	3.2	
moderate	922	26.4	21.3	11.2	<0.0001	404	20.7	20.3	12.3	0.0012	518	31.7	22.3	10.2	0.0001
high	490	16.7	9.8	4.8		278	18.6	10.9	7.1		212	14.9	8.7	2.7	
**Physical activity at school**															
low	87	2.3	1.4	1.9		46	2.4	1.8	1.8		41	2.2	0.9	2.0	
moderate	765	21.7	18.0	9.2	0.0017	330	16.0	17.7	9.9	0.0279	435	27.1	18.2	8.6	0.0009
vigorous	713	22.2	15.1	8.2		382	23.7	15.7	10.9		331	20.8	14.6	5.6	
**Physical activity at leisure time**															
low	149	2.8	3.1	3.6		76	2.5	3.3	4.2		73	3.0	3.0	3.1	
moderate	632	17.2	15.1	8.0	<0.0001	285	13.3	15.3	9.0	<0.0001	347	20.8	15.0	7.2	<0.0001
vigorous	786	26.4	16.2	7.6		397	26.3	16.6	9.5		389	26.6	15.7	5.8	

[1]Sample size may vary in variables due to missing data; [2]significance level of Chi^2 Pearson test.

International Journal of Environmental Research and Public Health

MDPI

Table S3. Distributions (%) of BMI category, central obesity and muscle strength by physical activity at school or at leisure time.

Variables	Gender-age-related BMI[2]			p-value[5]	Central obesity[3]	p-value	Muscle strenght[4]			p-value
	underweight	normal weight	overweight				lower	normal	higher	
**Sample size[1]**	145	980	368		182		147	893	148	74
**Sample percentage**	9.7	65.6	24.6		12.2		12.4	75.2	12.4	4.9
**Physical activity at school**										
low	3.8	58.2	38.0	<0.0001	20.3	0.0006	20.0	69.2	10.8	0.0003
moderate	10.5	61.1	28.3		14.4		14.1	76.9	9.0	
vigorous	9.4	71.3	19.3		8.9		9.6	74.0	16.4	
**Physical activity at leisure time**										
low	5.7	59.6	34.8	0.0003	21.1	<0.0001	16.2	75.7	8.1	0.2234
moderate	9.6	62.4	28.0		15.2		13.7	73.7	12.7	
vigorous	10.7	69.2	20.1		8.1		10.6	76.3	13.1	

[1]Sample size may vary in variables due to missing data; [2] gender-age-related BMI categorized according to international standards [35] as follows: underweight BMI<18.5 kg/m^2; normal weight BMI=18.5 to 24.9 kg/m^2; overweight BMI≥25 kg/m^2; [3]central obesity identified as waist-to-height ratio ≥0.5 [36]; [4]muscle strength identified as z-HGS (z-score handgrip strength) as follows: lower <−1 SD, normal −1 to 1 SD, higher >1 SD; [5]significance level of Chi^2 Pearson test.

Table S4. Association between physical activity during school, physical activity during leisure time and overweight, central obesity or muscle strength (OR$_A$)[1].

Variables	Overweight[2] (ref.: normal weight)	Central obesity[3] (ref.: lack of central obesity)	Higher muscle strenght[4] (ref.: normal muscle strength)
**Physical activity at school**			
moderate (ref. low)	0.78 (0.47; 1.29)	0.71 (0.39; 1.29)	0.73 (0.30; 1.77)
p-value[5]	0.3325	0.2590	0.4795
vigorous (ref. low)	0.45 (0.27; 0.75)	0.38 (0.20; 0.71)	1.38 (0.58; 3.25)
p-value	0.0020	0.0023	0.4629
**Physical activity at leisure time**			
moderate (ref. low)	0.81 (0.54; 1.22)	0.66 (0.41; 1.07)	1.45 (0.67; 3.14)
p-value	0.3114	0.0913	0.3399
vigorous (ref. low)	0.48 (0.30; 0.78)	0.31 (0.18; 0.51)	1.51 (0.71; 3.20)
p-value	0.0026	<0.0001	0.2807

[1]OR$_A$ = adjusted odds ratio (OR (95% confidence interval). Adjusted for gender, age (years), residence (categorical variable), Family Affluence Scale (points) and Nutrition Knowledge Score (points); [2] overweight and normal weight identified as gender-age-related-BMI according to international standards [35] as follows: overweight BMI≥25 kg/m^2, normal weight BMI=18.5 to 24.9 kg/m^2; [3]central obesity identified as waist-to-height ratio ≥0.5, lack of central obesity identified as waist-to-height ratio <0.5 [36]; [4]muscle strength identified as z-HGS (z-score handgrip strength) as follows: higher >1 SD, normal −1 to 1 SD; [5]significance level of Wald test.

International Journal of Environmental Research and Public Health

MDPI

Table S5. Association between physical activity during school, physical activity during leisure time and socioeconomics or demographics (ORA)[1].

Variables	Screen time (h/day)		Physical activity		Physical activity at school		Physical activity at leisure time	
	2 to < 4 (ref.: < 2)	≥4 (ref.: < 2)	moderate (ref.: low)	high (ref.: low)	moderate (ref.: low)	vigorous (ref.: low)	moderate (ref.: low)	vigorous (ref.: low)
**Gender**								
girls (ref. boys)	0.80 (0.64; 1.00)	0.62 (0.47; 0.82)	1.23 (0.87; 1.75)	0.67 (0.46; 0.99)	1.48 (0.94; 2.32)	0.93 (0.59; 1.47)	1.22 (0.85; 1.76)	0.88 (0.61; 1.27)
p-value[2]	0.0501	0.0008	0.2424	0.0439	0.0919	0.7556	0.2861	0.4924
**Age (years)**								
12 (ref.11 y)	0.92 (0.68; 1.24)	1.28 (0.86; 1.91)	0.86 (0.52; 1.41)	0.77 (0.45; 1.31)	0.95 (0.51; 1.79)	0.82 (0.42; 1.58)	0.87 (0.51; 1.47)	0.72 (0.43; 1.21)
p-value	0.5714	0.2214	0.5444	0.3327	0.8784	0.5460	0.6047	0.2188
13 (ref.11 y)	1.01 (0.62; 1.64)	1.61 (0.91; 2.86)	0.69 (0.33; 1.45)	0.48 (0.21; 1.08)	1.25 (0.45; 3.50)	1.03 (0.36; 2.94)	0.64 (0.29; 1.42)	0.47 (0.22; 1.02)
p-value	0.9684	0.0996	0.3304	0.0750	0.6659	0.9591	0.2683	0.0546
**Residence**								
urban (ref. rural)	0.86 (0.68; 1.08)	1.45 (1.08; 1.93)	0.70 (0.48; 1.02)	0.62 (0.41; 0.92)	0.86 (0.54; 1.37)	0.88 (0.55; 1.41)	0.65 (0.44; 0.96)	0.63 (0.43; 0.92)
p-value	0.1945	0.0122	0.0625	0.0171	0.5230	0.5923	0.0304	0.0174
**Family Affluence Scale**								
moderate (ref. low)	0.72 (0.54; 0.95)	0.70 (0.50; 0.98)	1.65 (1.12; 2.42)	1.99 (1.29; 3.07)	1.42 (0.86; 2.37)	1.85 (1.10; 3.12)	1.80 (1.20; 2.71)	2.03 (1.35; 3.04)
p-value	0.0220	0.0351	0.0112	0.0018	0.1709	0.0203	0.0045	0.0006
high (ref. low)	0.93 (0.68; 1.29)	0.77 (0.52; 1.15)	2.62 (1.54; 4.47)	3.53 (1.97; 6.31)	1.75 (0.91; 3.39)	2.70 (1.38; 5.31)	2.29 (1.33; 3.94)	3.06 (1.79; 5.24)
p-value	0.6704	0.1976	0.0004	<0.0001	0.0952	0.0038	0.0026	<0.0001

[1]ORA = adjusted odds ratio (OR (95% confidence interval). Adjusted for gender, age (years), residence (categorical variable), Family Affluence Scale (points) and Nutrition Knowledge Score (points), excluding the modelled variable from confounders set, respectively; [2]significance level of Wald test.

International Journal of Environmental Research and Public Health

MDPI

Table S6. Association between activity-inactivity patterns, ST, PA and overweight, central obesity or muscle strength (crude OR).

Variables	Overweight[1] (ref.: normal weight)	Central obesity[2] (ref.: lack of central obesity)	Higher muscle strenght[3] (ref.: normal muscle strength)
**Activity-inactivity patterns**			
highST-lowPA (ref. lowST- highPA)	4.86 (2.39; 9.91)	5.74 (2.27; 14.49)	0.97 (0.32; 2.94)
p-value[4]	<0.0001	0.0002	0.9539
lowST-lowPA (ref. lowST- highPA)	3.80 (1.83; 7.90)	6.67 (2.68; 16.56)	0.76 (0.21; 2.72)
p-value	0.0003	<0.0001	0.6652
highST-highPA (ref. lowST- highPA)	2.26 (1.15; 4.46)	1.95 (0.69; 5.50)	1.43 (0.63; 3.23)
p-value	0.0177	0.2029	0.3872
**Screen time (h/day)**			
2 to <4 (ref. <2)	1.82 (1.38; 2.40)	1.46 (1.01; 2.10)	1.18 (0.79; 1.75)
p-value	<0.0001	0.0422	0.4194
≥4 (ref. <2)	2.06 (1.49; 2.83)	2.20 (1.48; 3.27)	1.40 (0.88; 2.23)
p-value	<0.0001	<0.0001	0.1503
**Physical activity**			
moderate (ref.low)	0.50 (0.35; 0.73)	0.56 (0.36; 0.87)	1.39 (0.67; 2.89)
p-value	0.0003	0.0093	0.3739
high (ref.low)	0.28 (0.18; 0.42)	0.23 (0.13; 0.40)	2.20 (1.05; 4.61)
p-value	<0.0001	<0.0001	0.0367

ST - screen time; PA - physical activity. [1]overweight and normal weight identified as gender-age-related-BMI according to international standards [35] as follows: overweight BMI≥25 kg/m^2, normal weight BMI=18.5 to 24.9 kg/m^2; [2]central obesity identified as waist-to-height ratio ≥0.5, lack of central obesity identified as waist-to-height ratio <0.5 [36]; [3]muscle strength identified as z-HGS (z-score handgrip strength) as follows: higher >1 SD, normal −1 to 1 SD; [4]significance level of Wald test.

International Journal of
Environmental Research and Public Health

MDPI

Table S7. Association between activity-inactivity patterns, ST, PA and socioeconomics or demographics (crude OR).

Variables	Activity-inactivity patterns (ref.: lowST-highPA)			Screen time (h/day)		Physical activity	
	**highST-lowPA**	**lowST-lowPA**	**highST-highPA**	**2 to < 4 (ref.: < 2)**	**≥4 (ref.: < 2)**	**moderate (ref.: low)**	**high (ref.: low)**
**Gender**							
girls (ref. boys)	1.22 (0.67; 2.23)	1.62 (0.88; 3.00)	0.48 (0.28; 0.83)	0.80 (0.64; 1.00)	0.59 (0.45; 0.78)	1.26 (0.90; 1.78)	0.75 (0.52; 1.08)
p-value[1]	0.5130	0.1211	0.0089	0.0476	0.0001	0.1826	0.1212
**Age (years)**							
12 (ref. 11 y)	0.89 (0.40; 1.99)	1.82 (0.68; 4.90)	1.24 (0.59; 2.64)	0.93 (0.69; 1.24)	1.32 (0.89; 1.95)	0.84 (0.52; 1.38)	0.81 (0.48; 1.35)
p-value	0.7729	0.2321	0.5670	0.6059	0.1635	0.4992	0.4124
13 (ref. 11 y)	1.54 (0.47; 5.04)	1.85 (0.44; 7.86)	2.32 (0.82; 6.58)	0.96 (0.61; 1.49)	1.47 (0.85; 2.55)	0.90 (0.45; 1.81)	0.58 (0.27; 1.25)
p-value	0.4649	0.3949	0.1096	0.8416	0.1671	0.7763	0.1610
**Residence**							
urban (ref. rural)	2.61 (1.28; 5.33)	1.17 (0.63; 2.18)	0.99 (0.58; 1.67)	0.88 (0.70; 1.10)	1.38 (1.04; 1.83)	0.73 (0.51; 1.05)	0.63 (0.43; 0.92)
p-value	0.0083	0.6206	0.9579	0.2615	0.0243	0.0923	0.0171
**Family Affluence Scale**							
moderate (ref. low)	0.29 (0.15; 0.58)	0.47 (0.24; 0.94)	0.67 (0.34; 1.31)	0.71 (0.54; 0.94)	0.68 (0.49; 0.95)	1.59 (1.09; 2.33)	1.88 (1.24; 2.85)
p-value	0.0004	0.0308	0.2377	0.0181	0.0214	0.0171	0.0029
high (ref. low)	0.14 (0.05; 0.37)	0.12 (0.04; 0.37)	0.82 (0.40; 1.68)	0.93 (0.68; 1.28)	0.70 (0.48; 1.02)	2.72 (1.61; 4.60)	3.81 (2.18; 6.64)
p-value	<0.0001	0.0002	0.5800	0.6602	0.0641	0.0002	<0.0001

ST - screen time; PA - physical activity. [1]significance level of Wald test.
